# Intrapartum Ultrasound Guidance to Make Safer Any Obstetric Intervention: Fetal Head Rotation, Assisted Vaginal Birth, Breech Delivery of the Second Twin

**DOI:** 10.1097/GRF.0000000000000891

**Published:** 2024-10-18

**Authors:** Andrea Dall’Asta, Chiara Melito, Tullio Ghi

**Affiliations:** Department of Medicine and Surgery, Obstetrics and Gynecology Unit, University of Parma, Parma, Italy

**Keywords:** transabdominal ultrasound, persistent occiput position, labor dystocia, fetal attitude, head malposition

## Abstract

Intrapartum ultrasound (US) is more reliable than clinical assessment in determining parameters of crucial importance to optimize the management of labor including the position and station of the presenting part. Evidence from the literature supports the role of intrapartum US in predicting the outcome of labor in women diagnosed with slow progress during the first and second stage of labor, and randomized data have demonstrated that transabdominal US is far more accurate than digital examination in assessing fetal position before performing an instrumental delivery. Intrapartum US has also been shown to outperform the clinical skills in predicting the outcome and improving the technique of instrumental vaginal delivery. On this basis, some guidelines recommend intrapartum US to ascertain occiput position before performing an instrumental delivery. Manual rotation of occiput posterior position (MROP) and assisted breech delivery of the second twin are other obstetric interventions that can be performed during the second stage of labor with the support of intrapartum US. In this review article we summarize the existing evidence on the role of intrapartum US in assisting different types of obstetric intervention with the aim to improve their safety.

Intrapartum ultrasound (US) has been proposed with the aim to assist clinicians in different clinical scenarios of the labor ward practice. Observational^1–3^ and randomized trials^4–6^ have shown that intrapartum US is more reliable than clinical assessment in determining parameters of crucial importance to optimize the management of labor including the position and station of the presenting part.

Evidence from different research groups supports the role of intrapartum US in predicting the outcome of labor in women diagnosed with slow progress during the first^7–10^ and second^11–15^ stage of labor, and randomized data have demonstrated that transabdominal US is far more accurate than digital examination in assessing fetal position before performing an instrumental delivery.^5,6,16^ Instrumental vaginal delivery is the most common obstetric intervention performed during the second stage of labor to expedite delivery, and vacuum extractor is the most used type of instrument used worldwide for such aim. Intrapartum US has been shown to outperform the clinical skills in predicting the outcome and improving the technique of instrumental vaginal delivery.^11,16–22^ On this basis, some guidelines recommend^23–25^ the use of intrapartum US to ascertain occiput position before performing an instrumental delivery but also for the management of labor arrest and the objective diagnosis of malpresentation and asynclitism.^23^ Other obstetric interventions performed during the second stage of labor include manual rotation of occiput posterior position (MROP) and assisted breech delivery of the second twin. Each of them carries a risk of maternal and perinatal complications. As such, clinicians should be adequately trained in performing these interventions to minimize the risk of complications and ensure the safety of the dyad mother-fetus. US offers a visual support during the execution of these maneuvers. In this review article we summarize the existing evidence on the role of intrapartum US in assisting different types of obstetric intervention with the aim to improve their safety.

## BACKGROUND: EVOLUTION FROM CLINICAL TO SONOGRAPHIC ASSESSMENT

In the cephalic presenting fetus, the fetal position refers to the spatial relationship between fetal occiput and maternal pelvis.^26^ Head malposition is defined as any fetal position that is not occiput anterior (OA), being the occiput posterior (OP) position the most common. Although relatively common during the first-stage labor, fetal head malposition undergoes spontaneous rotation to OA in most cases; therefore, OP position at birth occurs in only 4% to 5% of women diagnosed with OP position during labor.^27–33^ With respect to occiput transverse (OT) position—that is, the second most common malposition—it is also transient accounting at birth for only 3% to 8% of the fetuses diagnosed with OT position during labor.^28,33,34^ Head malposition in the active phase of labor and particularly in the second stage is among the leading causes of labor arrest and obstetric intervention.^26,35^ Moreover, it is one of the strongest determinants of failed and complicated instrumental vaginal delivery.^6,36^ OP position has also been associated with peripartum complications such as obstetrical anal sphincter injury due to the compression of the larger diameter of fetal head against the anal sphincter,^28,33^ postpartum hemorrhage, chorioamnionitis, and birth trauma.^33,37,38^ The clinical diagnosis of head position in labor has been shown to be inaccurate in up to 1 in 2 cases,^3,5,30,39–45^ and importantly in approximately 20% of the women who are subsequently submitted to instrumental vaginal delivery.^5,6^ Transabdominal US represents the gold standard approach to determine the position of the fetal occiput^23,24^ through the visual demonstration of specific anatomical landmarks behind maternal pubis such as the orbits in the event of OP position, the fetal occiput, cerebellum and cervical spine in the event of OA position, and the horizontal direction of the midline of the fetal brain structures in the event of OT position.^23^ They can be identified by placing the probe transversely at the level of the suprapubic region. The transperineal approach on the axial plane represents an alternative for the diagnosis of head position, particularly when the fetal head is engaged in the birth canal, through the visualization of the anterior or posterior diverging appearance of the choroid plexuses toward the fetal occiput in the case of OA and OP positions, respectively, and the horizontal direction of the midline of the fetal brain in the event of OT position.

With respect to the head station, different sonographic indicators, including the angle of progression (AoP), head-perineum distance (HPD), midline angle (MLA), and head direction (HD), can be obtained using transperinal insonation.^46^ Midsagittal insonation allows to measure the AoP, which consists in the angle between the long axis of the pubic bone and a line from the lowest edge of the pubis drawn tangential to the deepest bony part of the fetal skull. Based on published data, the AoP indicates head engagement when its width is above 116 degrees.^47^ On the same plane of insonation, the head-symphysis distance (HSD) consists in the distance between the lower edge of pubic bone and the nearest point of the fetal skull along the infrapubic line.^48^ The HD is also measured on the midsagittal plane as the angle between the longest recognizable axis of the fetal head and the long axis of the pubic symphysis, classified categorically as “head down” (angle <0 degrees), “horizontal” (angle 0 to 30 degrees), and “head up” (angle >30 degrees).^47^ On the transperineal axial plane, the HPD consists in the shortest distance from the outer bony limit of the fetal skull to the perineum after compressing the perineal soft tissue against the pubic bone, and indicates head engagement at a length ranging between 35 and 38 mm.^17,49,50^ The MLA is the angle between the fetal head midline and the anteroposterior axis of the maternal pelvis. It has been associated with head station ≤+2 cm when its width is above 45 degrees and ≥+3 if the MLA width is below 45 degrees.^47^

## INTRAPARTUM ULTRASOUND AND FETAL HEAD MALPOSITION: PREDICTION OF VAGINAL DELIVERY AND VISUAL SUPPORT DURING MANUAL ROTATION OF OCCIPUT POSTERIOR POSITION

Fetal malposition may not impact on labor progression as most cases spontaneously rotate to OA and also those with persisting OP or OT position may negotiate the birth canal ending up with spontaneous vaginal delivery. On this basis, occiput position should not be sonographically ascertained on a routine basis in women with normal labor progression. In fact, Popowski et al^51^ evaluated in a RCT the use of routine US examination to determine fetal head position demonstrating that this policy did not improve labor management but increased the rate of obstetric intervention—both operative (33.7% vs 27.1%, RR 1.24, 95% CI 1.08-1.43) and cesarean (7.8% vs 4.9%, RR 1.60, 95% CI 1.12-2.28) deliveries—with no reduction of maternal and neonatal morbidity.

The Guidelines of the International Society of Ultrasound in Obstetrics and Gynecology recommend the use of intrapartum US as an adjunct to clinical evaluation in cases of labor dystocia in the first stage,^21^ as available evidence indicates that US is superior to digital examination for assessing fetal head station, progression, position, and attitude, and this has been shown to predict the outcome of labor.^21^ Head position has been shown to predict labor outcome in the context of labor dystocia in the first stage. Eggebo et al^9^ demonstrated an over 2-fold higher frequency of delivery by cesarean (38% vs 17% in non-OP position, *P*=0.01) in nulliparous women with OP position confirmed by transabdominal US. The indicators of head station have also been shown to predict the mode of delivery in the context of labor dystocia in the first stage. Eggebo et al^10^ found that HPD ≤40 mm [odds ratio (OR), 4.92; 95% CI 1.54-15.80], AoP ≥110 degrees (OR, 3.11; 95% CI 1.01-9.56), and nonocciput posterior position (OR 3.36; 95% CI 1.24-9.12) were independent predictors of vaginal delivery, whereas Brunelli et al^52^ demonstrated an independent association between the width of the AoP and vaginal delivery in cases diagnosed with labor dystocia in the first stage. More recently, our group has demonstrated that the sonographic assessment of fetal attitude combined with that of the fetal station and position improved the prediction of labor outcome in women diagnosed with dystocia in the first stage. Two transabdominal sonographic parameters have been described to quantify the fetal attitude, that is, the occiput-spine angle (OSA) for fetuses in OA or OT position and the chin-chest angle (CCA) in the event of OP position.^53–55^ The OSA is the angle formed by a line tangential to the occipital bone and a line tangential to the first vertebral body of the cervical spine, whereas CCA is the angle formed between one line through the longest axis of the sternum and another line through the skin overlying the mandible. The relationship between such transabdominal sonographic indicators of fetal attitude and the mode of delivery has been investigated in women with labor dystocia in the first stage showing an association between head deflexion (ie, a narrower OSA in fetuses in the OA and OT positions) and cesarean delivery.^55^ Consistently, Bellussi et al^56^ showed that a sonographic diagnosis of head deflexion at the beginning of the second stage represents an independent risk factor for cesarean delivery regardless the occiput position. More recently, Ramirez Zegarra et al^57^ evaluated the relationship between fetal attitude in fetuses in the OP position as evaluated by means of the CCA in the second stage of labor and persistent OP position at birth demonstrating an association between persistent OP position and a wider CCA (ie, fetal head deflexion) compared with the cases with spontaneous rotation of the fetal occiput (39.8±21.0 vs 54.9±26.2, *P*=0.007). The optimal CCA cutoff value discriminating rotating and nonrotating cases was 36.5 degrees, and the authors concluded that fetuses in OP position with a CCA below such CCA threshold might benefit from expectant management because of the high chance of delivery in the OA position without any intervention.

**FIGURE 1:**
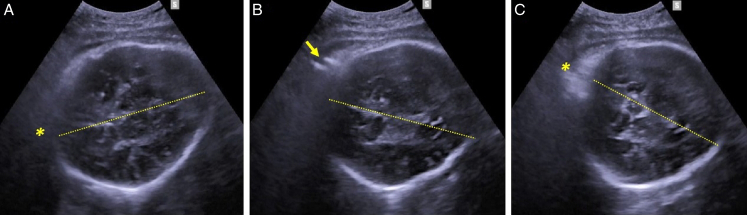
Transabdominal ultrasound demonstration of successful manual rotation from right transverse occiput position (8 o’clock) to right anterior occiput position (10 o’clock). A, Fetal occiput (*) at 8-o’clock position at the beginning of the procedure; (B) clockwise rotation of the fetal occiput and of the midline (dotted line) during the rotation procedure (fingers of the operator pointed by the arrow); and (C) fetal occiput (*) at 10-o’clock position at the beginning of the procedure.

Manual rotation of the fetal occiput (MROP) has been proposed as a prophylactic or therapeutic maneuver allowing the rotation of the fetal occiput from a posterior to an anterior position. The prerequisites for MROP are the OP or OT position with known position of the spine and unengaged fetal head. MROP has been proposed with the aim of reducing the rate of operative delivery, improving labor outcomes^58–62^ and preventing primary cesarean section during the second stage of labor.^63^ MROP can be performed by means of different approaches, one of the most popular being the Tarnier and Chantreuil method.^64^ It is performed with the patient lying in dorsolitotomy position and empty bladder. In first instance, in between uterine contractions, the operator places the right or left hand behind the fetal ear in the event of left or right position, respectively. Then, the patient is requested to start pushing following the onset of the uterine contraction, and at that point, the operator rotates the fetal head directioning the fetal occiput toward the anterior pelvis and maintaining the head in position until the next contraction. For the nondirect OP position, the direction of the rotational manuever is to be tailored in accordance with the spine position, that is, clockwise or counter clockwise with the fetal back on the right or on the left, respectively (Fig. 1). Anesthesia is beneficial for pain relief and makes the procedure more comfortable for the patient.

Prophylactic MROP is attempted in the early phase of the second stage of labor in absence of labor dystocia, while MROP is defined as “therapeutic” when the maneuver is performed to resolve head malposition in the context of a dystocic labor. With respect to prophylactic MROP, only the RCT of Blanc et al^65^ found a reduced risk of operative delivery in the intervention group compared with the standard group (29.4% vs 41.2%; *P*=0.047; differential [intervention-standard] [95% CI]=−11.8 [−15.7 to −7.9]; unadjusted odds ratio [95% CI]=0.593 [0.353-0.995]). Conversely, de Vries et al^66^ could not demonstrate a reduction in operative deliveries in the group of OT fetuses assigned to prophylactic manual rotation compared with that assigned to sham rotation (51% vs 50%; 95% CI −21 to 13; *P*=0.63). With respect to fetuses in OP position, Phipps et al^67^ also could not show differences in terms of obstetric intervention between the cases randomized to prophylactic MROP and those to sham rotation. Therefore, the effectiveness of MROP remains controversial,^68,69^ which may be explained by the fact that the success of the maneuver is dependent on the experience of the operator^70^ but also on the indication as well as the fetal attitude and station at the time of rotation.^57^ On this basis, one hypothesis is that only malpositions associated with labor dystocia may benefit from therapeutic MROP.^71^ Finally, intrapartum US has been shown to play a role not only in the diagnosis of malposition but also in assisting MROP via real-time guidance and confirming the final position of the fetal head after the procedure has been completed.^72^

## INTRAPARTUM ULTRASOUND BEFORE CONSIDERING OR PERFORMING AN INSTRUMENTAL DELIVERY

Instrumental vaginal delivery by vacuum extraction or forceps is performed with the aim to expedite delivery during the second stage of labor in the event of maternal exhaustion, maternal conditions associated to the need of avoiding pushing, labor arrest in the second stage, arrest of head descent, nonreassuring fetal heart rate,^73^ and accounts for approximately 5% to 15% deliveries in Western countries. Such obstetric intervention is acknowledged to be associated with maternal and perinatal complications including OASIs, postpartum hemorrhage,^37,38,74,75^ cephalohematoma, subgaleal or intracranial hemorrhage, skull fracture,^24,76–78^ and failed instrumental delivery leading to emergency cesarean section,^36,79,80^ the latter being associated with the highest rate of complications for the mother and the neonate.^81–88^ On this basis, the decision as to whether to accomplish delivery by instrumental vaginal delivery or by cesarean section is crucial to optimize the maternal and neonatal outcomes when an obstetric intervention is indicated during the second stage of labor.

As per International guidelines,^24,73^ instrumental delivery can be performed when the cervix is fully dilated, membranes are ruptured, the occiput position is known, and the fetal head is engaged. These 2 latter parameters (ie, fetal head position and level of engagement or station) are commonly evaluated by means of vaginal examination; however, the existing evidence suggests that such assessment is imprecise and with limited interoperator reproducibility irrespective of the level of expertise of the clinician.^4,89,90^

Grade “A” evidence supports the role of intrapartum US in the ascertainment of the occiput position before performing an instrumental delivery. On this basis, the ISUOG guidelines recommend in favor of the routine assessment of the occiput position before instrumental delivery,^23^ whereas the RCOG Guidelines recommend such assessment only in the event of uncertainty with respect to the clinically assessed occiput position.^24^ However, 3 randomized studies^5,6,91^ as well as 2 systematic reviews^92,93^ have failed to demonstrate a role of intrapartum US in improving the outcome of instrumental delivery. The Randomised Italian Sonography for occiput POSition Trial Ante vacuum (R.I.S.POS.T.A.)^6^ randomized women to vaginal examination (VE) + transabdominal US for assessment of the occiput position versus VE alone before instrumental vaginal delivery with the aim to investigate whether intrapartum US improves the outcome of instrumental delivery. The study was prematurely stopped for futility after randomizing less than a quarter of the estimated sample size, and this is likely to account for the fact that no difference between the randomization arms was noted in terms maternal and fetal outcomes; however, the study demonstrated a higher frequency of incorrect diagnosis of occiput position in the VE-only group (12.9% vs 4.5%, *P*=0.04). Such result was consistent with that of the Instrumental Delivery and Ultrasound (IDUS) randomized controlled trial by Ramphul et al,^5^ which also reported a lower frequency incorrect diagnosis of head position in the VE + US compared with the VE-only randomization arm (1.6% vs 20.2%, OR 0.06, 95% CI 0.02-0.19, *P*<0.001) with no difference between the 2 groups in terms of clinical outcomes. The latest published RCT investigating the role intrapartum US in improving the outcome of instrumental delivery compared with standard care was also suspended for futility and failed to demonstrate a clinical benefit in the ultrasound arm.^91^ Given the low frequency of the adverse outcome—that is, failed instrumental delivery—a large number of randomized cases is warranted to demonstrate a potential benefit of US as an adjunct to clinical examination for the ascertainment of the occiput position when compared with clinical examination only. In such context, the published RCTs are de facto underpowered to demonstrate a clinical role of intrapartum US in reducing the occurrence of failed instrumental delivery^5,6,91^ or of adverse maternal and perinatal outcomes. The R.I.S.POS.T.A. trial^6^ and the study by Barros et al^91^ were intended to be adequately powered to investigate whether the adjunct of intrapartum US is associated with a reduction of the frequency of failed vacuum delivery compared with standard clinical management of labor; however, both studies were prematurely stopped upon advice of a data safety monitoring committee based on exceedingly slow recruitment rate and an unexpectedly low frequency of the primary outcome. On this basis, the authors hypothesized that most practitioners commonly perform sonography before attempted vacuum extraction even in the absence of any evidence supporting its clinical benefit, and that randomization of a patient in the study was considered only when the fetal extraction was considered easy.^94^ Consistently, 2 meta-analyses assessing women undergoing instrumental vaginal delivery preceded versus nonpreceded by intrapartum US for evaluating the head position^92,93^ confirmed that ultrasound is more accurate than digital examination in establishing the fetal head position but could not demonstrate any benefit of the adjunct of intrapartum US in improving the maternal or perinatal outcome related to instrumental delivery.

Intrapartum US can also support clinicians in identifying women with a prolonged second stage of labor who may benefit from additional time of active pushing in the presence of reassuring fetal status due to a high chance of spontaneous vaginal delivery.^15^ Several studies investigating the role of intrapartum US in the context of second-stage dystocia have demonstrated its role in predicting the mode of delivery or the outcome of instrumental vaginal delivery.^11–14,17–19,21,95–97^ In a prospective study evaluating the chance of spontaneous vaginal delivery following a diagnosis of second-stage dystocia, the HSD and MLA have been shown represent the only parameters independently associated with spontaneous vaginal delivery.^15^ Kalache et al^11^ showed a 90% rate of successful vacuum extraction or spontaneous vaginal delivery in cases of second-stage dystocia featuring an angle of progression on or above 120 degrees, whereas Henrich et al^20^ demonstrated the association between the “head up” sign and successful vacuum delivery. Consistently, Kahrs et al^17^ reported a strong association between favorable indicators of head station and spontaneous vaginal delivery, successful instrumental delivery and short duration of vacuum extraction. More specifically, a 3.9% rate of cesarean delivery was recorded in cases featuring an HPD ≤35 mm compared with the 22.0% rate of women with HPD >35 mm (*P*<0.01); the duration of vacuum delivery was also shorter in women showing a short (ie, ≤25 mm) HPD compared with those with a long (ie, >25 mm) HPD. Masturzo et al^13^ assessed the role of the head direction in women with labor arrest during the second stage demonstrating a 4-fold higher risk of obstetric intervention in cases featuring a downward direction of the fetal head in the birth canal compared with those with an upward direction of the fetal head, and an over 2-fold higher frequency of obstetric intervention in the event of horizontal direction of the fetal head compared with the most favorable scenario represented by the upward head direction. HD and AoP may also predict the outcome of instrumental vaginal delivery. Henrich et al^20^ evaluated the role of the HD immediately before vacuum extraction reporting “easy” (5/11) or “moderately difficult” (6/11) vacuum extraction in the event of “head-up” direction, conversely only 1 of 6 vacuum extractions was reported as “easy” in the event of “head horizontal” or “head down” direction. With respect to the AoP, Sainz et al^21^ concluded that an AoP width AoP <105 degrees may identify cases of high risk of failure in instrumental deliveries. Bultez et al^18^ showed a lower median AoP width in the vacuum failure group compared with the cases with successful delivery [136.6 degrees (IQR, 129.8 to 144.1 degrees) vs 145.9 degrees (IQR, 135.0 to 158.4 degrees); *P*<0.01] and also reported a failure rate below 5% for AoP values above 145.5 degrees.

Among the other transperineal sonographic indicators of head station, the HPD has also been shown to represent the strongest predictor of the outcome of midcavity vacuum-assisted delivery^18^ and, more recently, vacuum delivery in fetuses in the OP position (unpublished data from our group). In a study by Nallet et al^95^ evaluating a selected cohort of women having clinically defined midcavity vacuum delivery, the reported failure rate of the procedure for the clinical stations 0 and +1 was 33.3% and 21.3%, respectively. In the same cohort, the reported rate of failed vacuum extraction was 12.9% in the event of HPD <50 mm. Furthermore, the HPD was the only variable independently associated with failed midcavity vacuum-assisted delivery at a station of 0 (adjusted OR 1.66; 95% CI 1.29-2.12; *P*<0.001), and the cutoff value discriminating between cases having successful versus failed vacuum delivery was 55 mm (of note, in this study, the HPD was measured without compressing the perineum, and this is why the predictive values obtained in this study are not comparable with those reported in the previous articles). In addition, unpublished data from our group suggest that in fetuses with sonographically confirmed OP position at the time of the decision to perform vacuum delivery show that the HPD represents the only parameter independently associated with failed extraction procedure. Of note, no differences in terms of AoP width were noted between the cases having a successful and those with failed vacuum delivery. The fact that a shorter HPD, but not a wider AoP, is associated with successful vacuum delivery can be explained by the fact that the pattern of descent of the fetal head in the birth canal differs between fetuses in OP and fetuses in the non-OP position.^98,99^ At midcavity, in OA fetuses the fetal head typically descends with a horizontal or upward orientation toward the pubis, and this is featured by a progressive widening of the angle of progression and a parallel shortening of the HPD. Conversely, in OP fetuses at midcavity, the fetal head shows with a downward orientation toward the sacrum, which results in a greater distance of the fetal head from the perineum (ie, HPD) compared with OA fetuses, whereas the width of the AoP is similar between the 2 groups. Such study conducted on a selected cohort of cases of confirmed OP position at the time of vacuum extraction also showed that in the event of sonographically confirmed OP position the rate of failed procedure is not dissimilar from that reported in unselected cohorts of cases in OP and non-OP positions.

The use of intrapartum ultrasound in the context of second-stage dystocia may allow to diagnose fetal head asynclitism.^100^ It is subclassified into anterior and posterior and can be diagnosed by means of transabdominal US by visualizing the midline of the fetal brain. In details, the diagnosis of anterior asynclitism is based on “anterior squint sign,” which consists in the demonstration of the posterior displacement of the midsagittal suture and the visualization of the anterior orbit only; conversely, posterior asynclitism is characterized by the “posterior squint sign,” which consists in the anterior displacement of midline of the fetal brain.^101–103^ During the second stage of labor, the US diagnosis of asynclitism is preferably performed transperineally on the axial plane. The “asynclitic midline sign” consists in the visualization of the midline not equidistant from the parietal bones.^100,104^ More specifically, in the event of anterior asynclitism, the midline of the fetal brain is seen close to the sacrum as the visualization of the midline equidistant from the parietal bones can be achieved by tilting the probe downward^104^ and moving it close to the pubic symphysis^100^; conversely, in posterior asynclitism, the midline of the fetal brain is seen close to the pubic symphysis, and the visualization of the midline equidistant from the parietal bones can be achieved by tilting the probe upward^105^ and moving it toward the body of the perineum. Persistent asynclitism may be associated with labor dystocia in the first or second stage, and increase the rate of obstetric intervention and failed instrumental delivery.^100,101^ Hung et al^106^ examined the prevalence and outcome of asynclitism in the second stage of labor showing a higher prevalence of asynclitism in non-OA compared with OA position (53% vs 6.7%, *P*<0.01), with no difference in terms of frequency of obstetric intervention between cases with and without asynclitism (42.9% vs 26.9%, *P*=0.22). In the same study, the delta HPD during the pushing efforts was narrower in cases of asynclitism compared with those with no evidence of asynclitism (0.68 vs 0.91 cm, *P*=0.01). Posterior asynclitism is acknowledged as a limiting factor for the vaginal delivery of a normally grown term fetus; hence, when such diagnosis is made in a dystocic labor, the option of instrumental delivery is to be considered with caution.^100^

Beyond the identification of the actual occiput position before performing an instrumental delivery and the prediction of the mode of delivery in the context of second-stage dystocia, intrapartum US has been proven to assist the physician in the application of the obstetric instrument. Suboptimal vacuum cup placement has been documented in approximately 40% of instrumental vaginal delivery.^107^ Ramphul et al^36^ found a higher maternal hospital stay (adjusted OR 2.28, 95% CI 1.30-4.02), neonatal trauma (adjusted OR 4.25, 95% CI 1.85-9.72), use of sequential instruments (adjusted OR 3.99, 95% CI 1.94-8.23), and cesarean section for failed instrumental delivery (adjusted OR 3.81, 95% CI 1.10-13.16), following an incorrect placement of the instrument (vacuum or forceps). The parameters associated with suboptimal placement were head malposition (OR 2.44, 95% CI 1.62-3.66), midcavity station (OR 1.68, 95% CI 1.02-2.78). Intrapartum US assists clinicians in determining the actual occiput position, hence in identifying the flexion point where to appropriately place the vacuum. In the event of head malposition such as OP the flexion point is located at a higher level and more posteriorly in the birth canal and for this reason is more difficult to be adequately assessed by means of vaginal examination. Wong et al^16^ evaluated the accuracy of vacuum cup placement randomizing women to receive digital examination alone or combined with transabdominal ultrasound assessment before accomplish the procedure: the accuracy of the vacuum cup placement was improved in the ultrasound group, which was featured by cup placement closer to the flexion point, albeit with no reduction in maternal and fetal morbidities.

With respect to forceps delivery, intrapartum US has been proposed to assist clinicians in placing the blades of the forceps and performing rotational instrumental delivery with Kielland forceps. Hinkson et al^22^ performed real-time suprapubic ultrasound during rotational forceps deliveries in cases of arrest of labor in the second stage showing successful rotation to occiput anterior position of all cases submitted to intrapartum US and no case of failed instrumental delivery. The proposed benefits of US assistance included the correct placement of the instrument, the avoidance of trauma on the fetal head, and the confirmation of the correct rotation of the head, in real-time, to support the change in the position of the forceps handles.

**FIGURE 2:**
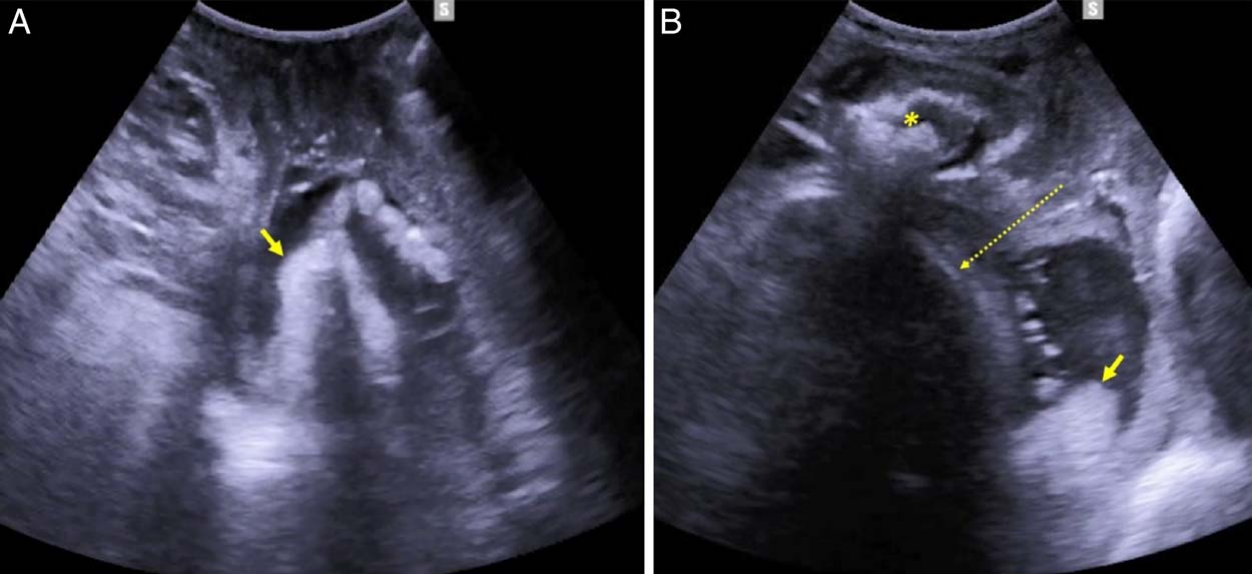
Transperineal ultrasound demonstration of compound hand-cephalic presentation. A, On the axial plane the hand (arrow) is seen preceding the fetal head in the birth canal. B, Midsagittal view demonstrating the relation between the hand (arrow), the pubic symphysis (*), and the fetal head (dotted arrow).

Finally, intrapartum US has been shown to contribute in diagnosis and management of compound hand-cephalic presentation. This refers to an extremity—most commonly the upper limb—prolapsed alongside the presenting part which is commonly diagnosed at clinical examination (Fig. 2). In most cases, the prolapsed part withdraws with labor progression; if this does not occur, the fetal upper limb should be pushed upward by the operator, whereas fundal pressure is applied to favor the head descent.^108^ Intrapartum US has been shown to be highly accurate in confirming the diagnosis and assisting in management because the clinician can be facilitated in the maneuver of reducing the prolapsed limb due to the exact knowledge of which limb is in front of the presenting part.^109^

## INTRAPARTUM ULTRASOUND TO ASSIST FETAL EXTRACTION IN THE EVENT OF BREECH DELIVERY OF THE SECOND TWIN

Another recently described application of intrapartum ultrasound consists in assisted breech delivery of the second twin.

The optimal mode of delivery in twins is yet to be defined. Vaginal delivery in twins had been associated with a list of complications particularly related to the delivery of the second twin and including malpresentation, cord prolapse, placental abruption and increased morbidity.^110^ The Twin Birth Study was the first and to date is the only available RCT evaluating labor outcomes of twin pregnancies with cephalic presenting first twin randomized to planned vaginal delivery versus cesarean section.^98^ The study could not demonstrate any difference in the primary outcome—that is, a composite of fetal or neonatal death or serious neonatal morbidity—in cases randomized to planned cesarean section versus in those with planned vaginal delivery. More recently, the French prospective cohort from the JUMODA trial showed an over two-fold higher composite neonatal mortality and morbidity in twins delivered by planned cesarean section compared with planned vaginal delivery (5.2% compared with 2.2%; odds ratio [OR] 2.38, 95% CI 1.86-3.05) concluding that planned cesarean between 32 and 37 weeks of gestation might be associated with increased composite neonatal mortality and morbidity.^99^

On this basis, the American College of Obstetrics and Gynecology, the National Institute for Health and Care Excellence, the International Federation of Gynecology and Obstetrics, and the Society of Obstetricians and Gynecologists of Canada recommend vaginal delivery if the first twin has a cephalic presentation, regardless of the presentation of the second twin.^111–115^

In the event of noncephalic presenting second twin, internal version maneuvers followed by a breech extraction are recommended to accomplish its vaginal delivery.^111^ A prerequisite for such maneuver is the identification of the most anterior lower limb, which represents the first structure that needs to be extracted from the birth canal. Of note, the integrity of the amniotic membranes is crucial to prevent umbilical cord prolapse and allow improved mobility while performing the internal version maneuver.^111^ Clinically, the discrimination between the two legs could be done recognizing the fetal hallux. Intrapartum US can help clinicians in assisting in real-time the hand of the operator reaching the most anterior leg. As such, it is also reasonable to speculate that the sonographic identification of the most anterior lower limb by means of intrapartum US may reduce the risk of rupture of the amniotic membranes compared with the “blinded” manual identification of the lower limbs. A recent publication from our group showed a case of intrapartum US-assisted the internal version maneuver with intact membranes and the breech delivery of a transverse lying second twin, thus supporting the concept that intrapartum US may simplify a potentially challenging maneuver.

## CONCLUSIONS

To conclude, albeit in the absence of grade A evidence, several observational studies and reports support the concept that intrapartum US has the potential to assist clinicians considering or performing any obstetric intervention during the second stage of labor. Of note, such potential is not limited to the prediction of the mode of delivery in the context of labor dystocia and in assisting and predicting the outcome of instrumental delivery but include also MROP and fetal extraction of breech second twin may benefit from the adjunct of US to the conventional clinical management.
